# The Role of Water in Protein–Ligand Binding: Decisive Factor, Annoying Feature, or Indispensable Control Element: In Any Case, a Major Challenge

**DOI:** 10.1002/cmdc.70383

**Published:** 2026-07-13

**Authors:** Gerhard Klebe

**Affiliations:** ^1^ Institute of Pharmaceutical Chemistry Philipps University Marburg Marburg Germany

**Keywords:** chemistry, implicit solvation, ligand, molecule, protein‐ligand binding, solvation, solvation shell

## Abstract

Evolution began in water; it's not surprising that water had a profound influence on protein–ligand complex formation. However, water's contribution to binding is low, perhaps up to ±5 kJ/mol for displacement or recruitment. When affinity is factorized into enthalpy and entropy, a large scatter of both is observed, especially when both mutually compensate. Since this also applies to water, its impact can easily be overlooked and go unnoticed, especially when only affinity data are available. Nevertheless, water can enhance ligand affinity when a well‐designed solvation shell assembles around the formed protein–ligand complex. Examples are discussed of pockets exhibiting varying water density in uncomplexed proteins, or transient pockets that only open with ligand binding. Are these pockets solvated before the ligand enters, or can the ligand penetrate without allowing water molecules to temporarily fill the pocket? Rules are needed to help medicinal chemists select the best optimization strategy while considering water's impact.

## Graphical abstract

Water influences ligand binding to a protein. Replacing, picking up, or modulating water dynamics restructures the water arrangement and affects the enthalpic and entropic signatures of binding. Whether the two properties add up or cancel each other out determines the expected ligand affinity enhancement.

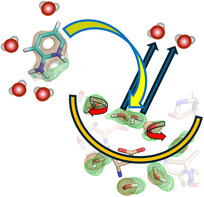

## 1 Introduction

In 2011, this journal asked me who I would like to be if I could be anyone else for a day [1]. Jokingly, I replied that I would like to be a water molecule to understand my role in binding drugs to proteins. Of course, this wish could not be realized. Nevertheless, I believe that understanding the role of water in the drug‐receptor binding process is essential to creating more effective and predictive drug design tools. Why is that, and have we come any closer to understanding the role of water molecules in drug discovery? In the following essay, these aspects will be discussed from the author's personal perspective. This contribution does not attempt to provide an overview or comprehensive review of the current state of research in this field, either with respect to experimental results or tool developments for computational simulations. Such an objective would be beyond the scope of this essay. Readers looking for such an overview are referred to excellent review articles published in recent years [2, 3, 4, 5, 6, 7].

Biological evolution began in water. Life developed in this environment, and many years passed before life left the water and moved to land. Therefore, it is not surprising that water has had a profound influence on the molecules that govern life [8]. Our own bodies are filled to 60% with water. For the past 20 years, our research group has collected as much experimental information as possible about the impact of water on drug binding. We primarily used isothermal titration calorimetry (ITC) and structural analyses, complemented by molecular dynamics simulations to validate the relevance of an applied simulation model. The structural studies included high‐resolution X‐ray crystallography, neutron diffraction, EPR, and NMR spectroscopy [9].

### 1.1 Collecting Information on Water

What did we learn? An inspection of the database of resolved protein structures revealed that a significant percentage of the protein‐ligand complexes deposited in the PDB demonstrate the involvement of water molecules in ligand binding. When analyzing such data, it is important to consider that water molecules only weakly scatter X‐rays and are often mobile. They exhibit high residual mobility even in the bound state, which reduces their scattering power and makes reliably detecting them difficult. This problem is less significant with neutrons, used for diffraction, because hydrogen and deuterium (heavy water) strongly contribute to the diffraction signal. Scattering occurs at the nuclear potential, and both hydrogen isotopes are strong scatterers. They can even be distinguished, which gives rise to many remarkable experiments. Thus, neutron scattering allows us to obtain a more reliable and detailed picture of water molecules, suggesting that neutron diffraction is a superior tool for studying biological macromolecules.

So why not use neutron scattering as the standard technique to study biological molecules? The problem is scattering power [10, 11, 12]. Laboratory X‐ray sources provide 10^6^ to 10^11^ photons per second, resulting in data collection times of hours to days for crystals sized 20–300 µm. Modern synchrotrons increase the flux by four to six orders of magnitude, enabling the collection of complete datasets in a matter of seconds to minutes for crystals as small as a few micrometers. Free‐electron lasers deliver 10^12^ to 10^13^ photons per pulse with extreme peak brilliance, enabling millisecond‐scale serial data collection. In contrast, neutron sources provide 10^13^ to 10^16^ neutrons per cm^2^ and per second, but they require millimeter‐scale crystals. This leads to measurement times of hours to days at reactors and is typically 5–10 times faster at spallation sources. Consequently, the number of high‐resolution neutron diffraction studies deposited in the PDB is still very scarce (ca. 0.1% of all determined crystal structures), and the technique will likely remain limited despite its amazing power.

Thus, we must work with the available X‐ray data in the PDB database. Unfortunately, the lower the resolution of the collected data, the more water molecules will be missed or remain undetected when studying a protein–ligand complex and no information about the orientation of the protons becomes transparent. For these reasons, we limited ourselves to data collected at a resolution of better than 1.5 Å.

Some readers may wonder, if collecting experimental data on water is so difficult, why not use computational tools to study its impact on protein‐ligand binding? These tools are typically based on molecular dynamics (MD) simulations using well‐established force fields [4, 5, 6, 7, 13, 14, 15]. However, it is unclear whether the applied force fields are sufficiently well‐parameterized to correctly reproduce the properties of water, especially considering the significant dipole moment of water molecules and the resulting induced polarization effects. For the intended studies, the individual water molecules must be handled explicitly. The polarization phenomena involved depend on the local dielectric conditions. They can be assigned differently within the protein's interior, along its outer rim, or in individual pockets. Therefore, we must distinguish whether the considered water molecules are confined in a sealed hydrophobic or hydrophilic pocket, or if they are still in contact with the surrounding bulk water phase. Additionally, we must consider extensive hydrogen‐bonding networks among water molecules, protein residues, and ligand functional groups [16, 17, 18]. We cannot assume that the water density in an uncomplexed binding pocket is the same as or comparable to that of the surrounding bulk water phase. Furthermore, water molecules can adopt different protonation states in binding pockets [19]. Ultimately, chemistry and biology are experimental disciplines that are typically studied, simulated, and validated through models based on theoretical concepts. Therefore, any correlation based on these concepts that is used to predict the properties or behavior of a biological or chemical system must be validated through experimentation to ensure its correctness. Otherwise, the correlation is considered falsified. We typically study series of potential lead or drug candidates and attempt to predict their properties using a given model. In this context, identifying “outliers” in the model is essential. Outliers may result from inaccurate measurements. In this case, the experiments must be repeated until sufficient reproducibility is achieved. However, outliers may also suggest that the model's assumptions are incorrect, the applied tools are inappropriate or deficient, or our understanding of the matter is still incomplete. In this case, the theoretical model is falsified through experimental validation, necessitating the creation of an improved model.

### 1.2 Water Impact on Binding Thermodynamics

Thermodynamically, binding affinity is a Gibbs free energy (Δ*G* ^0^), which is composed additively of an enthalpic contribution (Δ*H* ^0^) and an entropic contribution (*−T*Δ*S* ^0^). In lead optimization, one goal is to enhance binding affinity by changing both properties in the same direction towards more negative values. This shifts the Gibbs free energy of binding (Δ*G* ^0^) towards more negative values, thus enhancing affinity. However, in many cases, the two properties work against each other. This can easily nullify the improvements in affinity during ligand optimization because the effects of optimizing one property are offset by the other property's compensating effect. The following consideration may help illustrate this phenomenon. High residual mobility at the binding site is entropically favorable since, upon transitioning from the solvated to the bound state, fewer degrees of freedom are lost. However, high residual mobility at the binding site hinders the formation of beneficial and enthalpically favored hydrogen bonds. This simple example gives an idea of why enthalpy and entropy can work against each other. Of course, a binding event is much more complex and involves many more factors. Apart from the loss of degrees of freedom and hydrogen bonds, aspects such as solvation, preorganization, water displacement or recruitment, polarization, or protein adaptations matter [3]. The interplay between enthalpy and entropy is called enthalpy/entropy compensation, which has a significant impact on the success of affinity optimization [20, 21, 22, 23]. There is no physical relationship that defines mutual enthalpy/entropy compensation in protein‐ligand complexes. Evidence for its importance in protein‐ligand binding is based on empirical observations. Correlating experimental data shows, however, that the scatter which improves Δ*G* ^0^ during optimization ranges from millimolar to nanomolar affinity over about 30 kJ/mol. However, the scatter due to enthalpy/entropy compensation spreads across a range about six times larger [9, 24]. Therefore, a successful optimization strategy must keep enthalpy/entropy compensation under control.

Furthermore, the thermodynamic analysis of protein‐ligand complexes suggests that the impact of the displacement or recruitment of a water molecule on ligand binding is weak, perhaps up to ±5 kJ/mol in affinity or Gibbs free energy of binding (Δ*G* ^0^) [9]. Again, the partitioning of enthalpy and entropy reveals a scatter approximately six times larger than that of Δ*G* ^0^, where enthalpy and entropy show opposing contributions to Δ*G* ^0^. Therefore, the mutual compensation of the two properties also leads here to significant enthalpy/entropy compensation [9].

Only experiments that partition Δ*H* ^0^ and *−T*Δ*S* ^0^ can disclose the impact of enthalpy/entropy compensation on lead optimization. Unfortunately, most medicinal chemistry studies only record affinity data (Δ*G* ^0^) and not detailed enthalpy/entropy signatures. This often makes optimization strategies based solely on Δ*G* ^0^ data difficult. Since water can significantly impact enthalpy/entropy compensation but only slightly affect Δ*G* ^0^ (as the opposing enthalpy and entropy contributions cancel each other out), its contribution can easily go unnoticed. Again, the mutual compensation of these two properties leads to significant enthalpy/entropy compensation. Accordingly, the water also contributes to this compensation [9].

**FIGURE 1:**
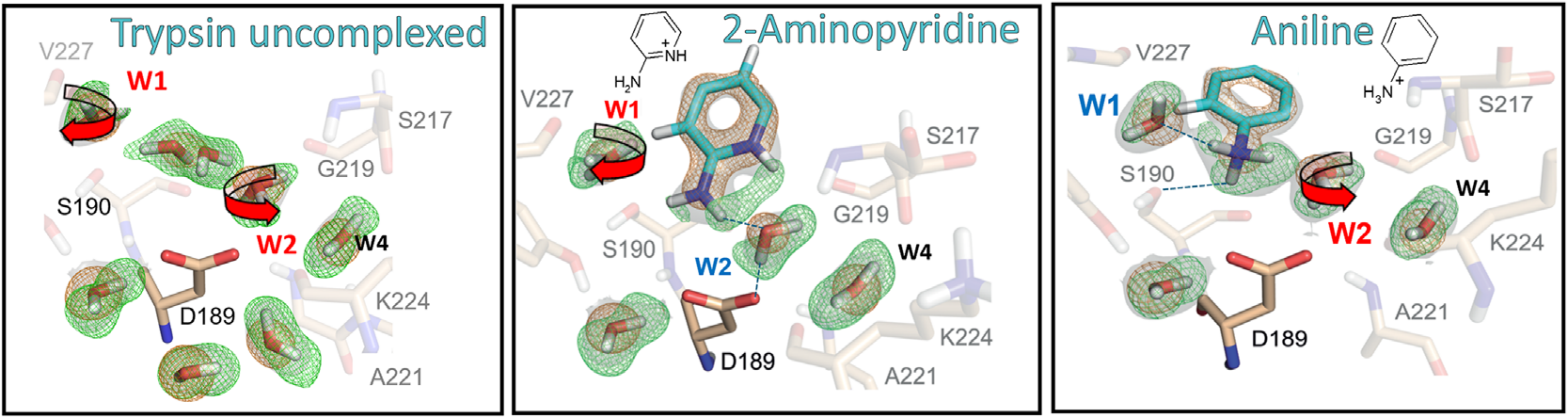
Binding pocket of trypsin, analyzed by a combined neutron and X‐ray diffraction study. The uncomplexed S_1_ pocket is shown on the left and several water molecules are observed. Of these W1 and W2 (red) show enhanced residual mobility in the pocket (more than two hydrogen sites are populated). Once 2‐aminopyridine is bound (center) two water molecules, originally located next to the carboxylate group of Asp 189, are displaced by the ligand; W2 adopts ordered geometry (blue, density shows two hydrogen positions) whereas W1 (red) still shows residual mobility. If aniline is accommodated (right), W1 adopts order geometry (blue), whereas W2 exhibits pronounced residual mobility in the pocket (red). Protein shown in stick display mode, carbon atoms in pale, carbon atoms of the ligands in cyan. The scatter density around the hydrogen atoms of the heavy water molecules is contoured by a green mesh, the water oxygen atoms by the X‐ray diffracted electron density with an orange mesh.

The following study of trypsin‐ligand complexes using neutron diffraction sheds some light on the impact of water on ligand binding (Figure 1). The study revealed that some water molecules from the uncomplexed protein become mediators of hydrogen bonds with the ligand, while others are displaced [25]. Surprisingly, some of the remaining water molecules alter their dynamic properties in response to the accommodated ligand, transitioning from a fixed to a mobile state and vice versa. These changes differ from one bound ligand to another and will likely modulate the thermodynamic profile of the individual water molecules. Consequently, the overall thermodynamic signature will shift from a more enthalpy‐dominated to a more entropy‐dominated profile. This shift then contributes to the mutual compensation of enthalpy and entropy during complex formation. The experimental evidence collected for the modulated dynamic behavior of the water molecules is based on differences in the observed scatter density. In addition to these observable local water dynamics, the residence time and exchange rate of water molecules in a binding pocket are important. However, these are much more difficult to trace experimentally. Molecular dynamics simulations suggest that the time intervals that water molecules remain at a given hydration site differ depending on the local environment. These exchange dynamics are likely equally crucial for the functional impact of water [26, 27].

### 1.3 Enthalpy/Entropy Compensation—A Pain in the Neck

Nevertheless, the enthalpy/entropy compensation can be both a curse and a blessing in our science. It is annoying that the potential contributions of water molecules can be easily overlooked due to this compensation. However, it may also explain why virtual screening based on docking and affinity scoring still works reasonably well without explicitly considering water molecules, simply because their influence on affinity is minor due to compensating effects. Undetected water molecules in docking, however, may lead to false binding poses of bound ligands, resulting in incorrect predictions in the subsequent ligand optimization strategy [28]. Another obstacle may arise when applying quantitative structure–activity relationships (QSAR) [29]. QSAR is very popular and frequently applied in drug optimization. It correlates relative affinity differences with molecular descriptors to establish a design hypothesis for further ligand optimization. From a pragmatic point of view, any molecular descriptor could be used. However, there is a risk that descriptors will be selected which correlate well with affinity trends in the ligand series used for training because they reflect by chance the trends in the series correctly, rather than because they are the correctly selected relevant properties. The following example illustrates the point of such a chance correlation. Many modelers observed that molecular weight correlates with affinity differences within their dataset. However, molecular weight is not a useful descriptor for further design because ligand improvements usually increase molecular size and weight anyway. While this example is obvious, such chance correlations are often more difficult to trace. This is particularly true in cases where water molecules contribute to affinity because the impact of water can easily be masked by other descriptors [29, 30].

### 1.4 Surface Solvation Shell Improves Ligand Binding

Now, back to the impact of water on ligand binding. One effect we observed that can be exploited in molecular modeling is the residual solvation of bound ligands in a binding pocket. Terminal parts of ligands often occupy open, bowl‐shaped, or crevice‐shaped binding pockets that open to the exterior of a protein. There, the ligand protrudes into the surrounding bulk water phase, and a novel solvation shell assembles around the complex [31, 32]. Depending on the structural features of the exposed ligand portion, the newly assembled interface can form an ideal surface water network or can lead to local perturbations of the surrounding water environment. In this case, the geometry of the water shell will remain incomplete with local misfits. This can reduce ligand binding affinity. Ideally, the water confinement around an exposed hydrophobic group forms a perfect surface water network, thereby significantly increasing the affinity of the bound ligand. This feature can also be exploited to enhance ligand selectivity.

**FIGURE 2:**
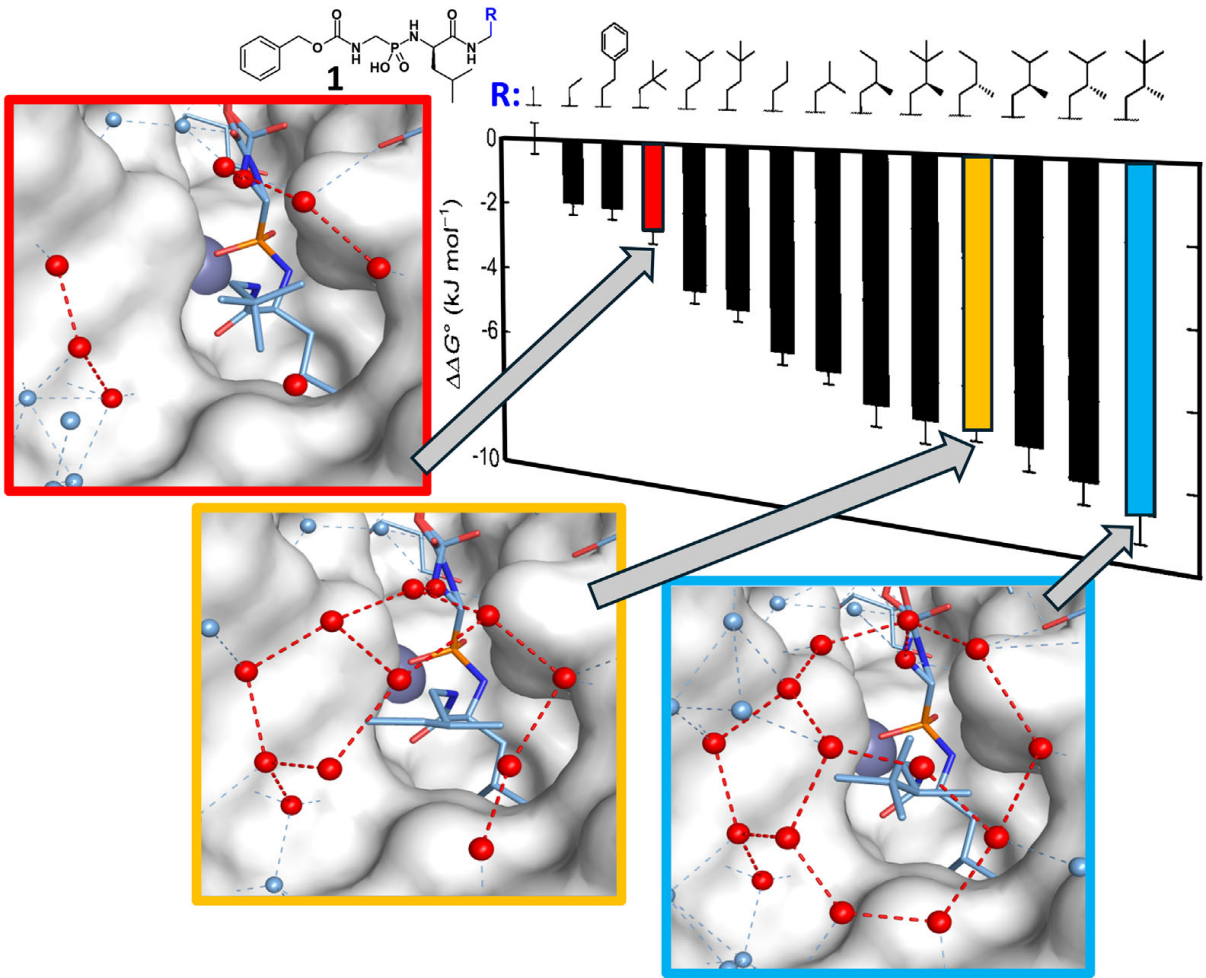
Binding of a series of ligands with the parent scaffold **1** to the enzyme thermolysin. The P_2_’ side chains of the studied ligands place their aliphatic substituent R in the open bowl‐shaped S_2_’ pocket. Towards the surrounding bulk water phase, a solvation shell is formed that wraps around the exposed side chain and shields increasingly the ligand by a network of interconnected chains of hydrogen bonds. With increasing completeness of the covering water polygon, the affinity of the ligand improves by a factor of 50. Affinity differences are listed as ΔΔ*G* ^0^, relative to the first complex of the series. The protein surface is displayed as white solvent‐accessible surface, ligands shown as stick model, water molecules as red spheres.

We studied a series of thermolysin inhibitors that were exposed to the exterior of the enzyme's S_2_′ pocket (Figure 2). Decorating the parent ligand scaffold **1** with an ideal fitting side chain resulted in a 50‐fold increase in affinity [32]. The improvement was achieved by forming energetically favorable five‐ to eight‐membered water polygons that fuze by chains of interconnected hydrogen bonds. The chains wrap around the exposed ligand portion like a hood. Molecular dynamics (MD) simulations of the water environment helped designing a ligand side chain that best fitted the formed surface water network [32, 33]. However, attempts involving polar and charged groups extending beyond the binding pocket resulted in an affinity loss [34]. This is likely because significant electrostatic perturbations of the surface water network occur, canceling out the desired enhancing effect of the formed water shell hood.

### 1.5 Are Pockets Uniformly Solvated, Similar to the Bulk Phase

Ligand binding occurs in protein pockets. Prior to ligand accommodation, these pockets may be solvated. However, the water density in uncomplexed pockets can differ greatly from pocket to pocket, as well as from the surrounding bulk water phase. Stephen Homans first proposed the idea that uncomplexed binding pockets exhibit varying water densities [35]. Even more complicated are the transient pockets that only open with ligand binding. Are these pockets solvated before the ligand enters, or does the ligand penetrate without allowing water molecules to temporarily fill the pocket? In this context, two extreme models are discussed: induced fit and conformational selection [36]. In the induced fit model, the pocket only opens once the ligand approaches the pocket and triggers the opening process. In the second model, open and closed conformations of the pocket are in dynamic equilibrium, and the ligand selects the pocket with the correct shape for its binding. Probably, there is a continuous transition between these two extremes; however, how do these models affect water penetration and ligand binding affinity?

**FIGURE 3:**
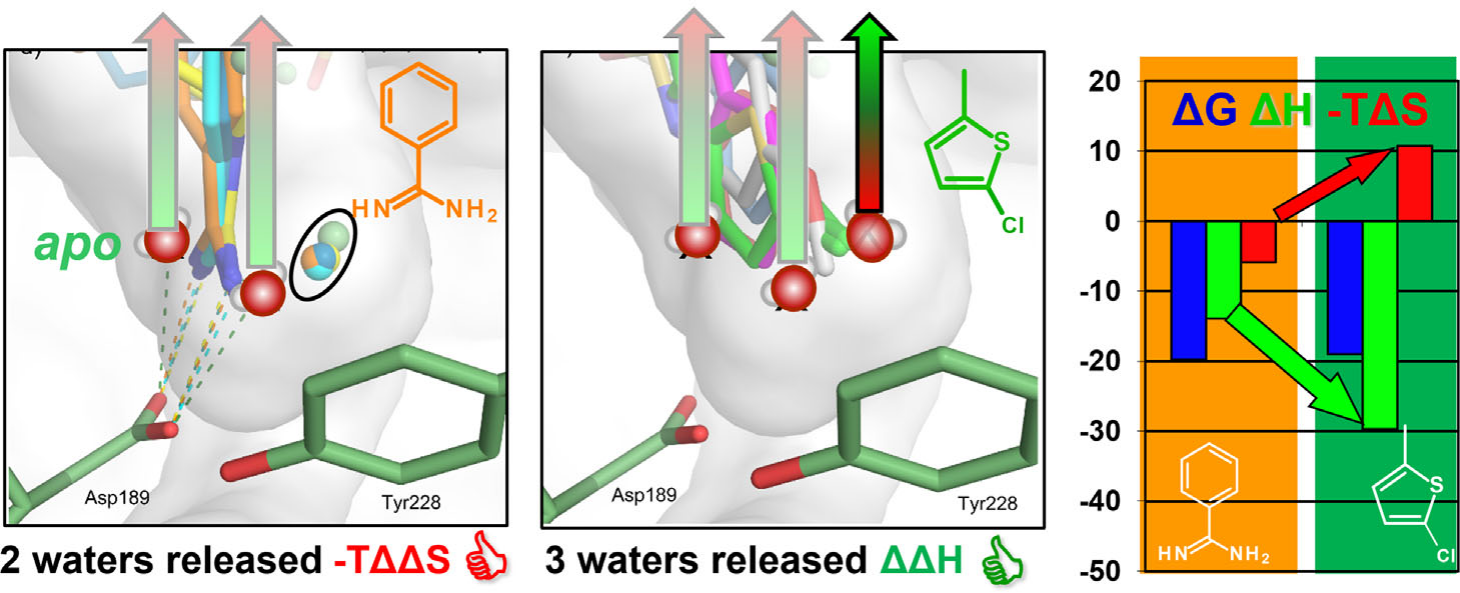
Binding of benzamidine (orange) and 2‐chloro‐5‐methyl‐thiophen (green) to the S_1_ pocket for thrombin. The displacement of two seemingly well‐ordered water molecules from the pocket by benzamidine results in a more entropy‐favored signature compared to the chlorothiophen that displaces three water molecules from the site (the third additional water molecule highlighted by the arrow). The third additional water molecule shows high residual mobility (cf. structure determined by neutron diffraction with the isostructural trypsin) shifts the overall profile towards a more enthalpy‐driven signature (cf. histograms on the right).

Let's consider first stable pockets. Both thrombin and trypsin have well‐defined recognition pockets on their surfaces that can accommodate peptides to be cleaved. The S_1_ pocket contains several water molecules that can be displaced by a bound ligand [37]. Once these in the uncomplexed structure ordered water molecules are displaced by a ligand, a binding profile with favorable entropy contribution is recorded (Figure 3). Upon release, the water molecules lose enthalpically favored interactions but gain degrees of freedom in the bulk water phase, which increases entropy overall. Above Tyr 228, however, there is a water molecule that exhibits high residual mobility in the uncomplexed state. This water molecule is already entropically strongly favored in the protein‐bound state; therefore, its additional displacement by a ligand results in an enthalpically favored binding signature.

In thermolysin, we found that the S_1_’ pocket is virtually unsolvated; however, its shape would allow it to host up to five water molecules [38]. The protease accommodates the hydrophobic side chain of a peptide substrate in its S_1_’ pocket and strongly favors Leu, Ile, and Phe over Val, Ala, and Gly. Remarkably, the binding signature of substrate‐analog inhibitors shows a strong enthalpic signal. This signature can be explained by the crystallographic observation that the pocket is virtually unsolvated, meaning no or only a little price for desolvation of the pocket has to be paid. As a result, an overall enthalpic profile remains.

### 1.6 Are Transient Pockets Temporarily Solvated

**FIGURE 4:**
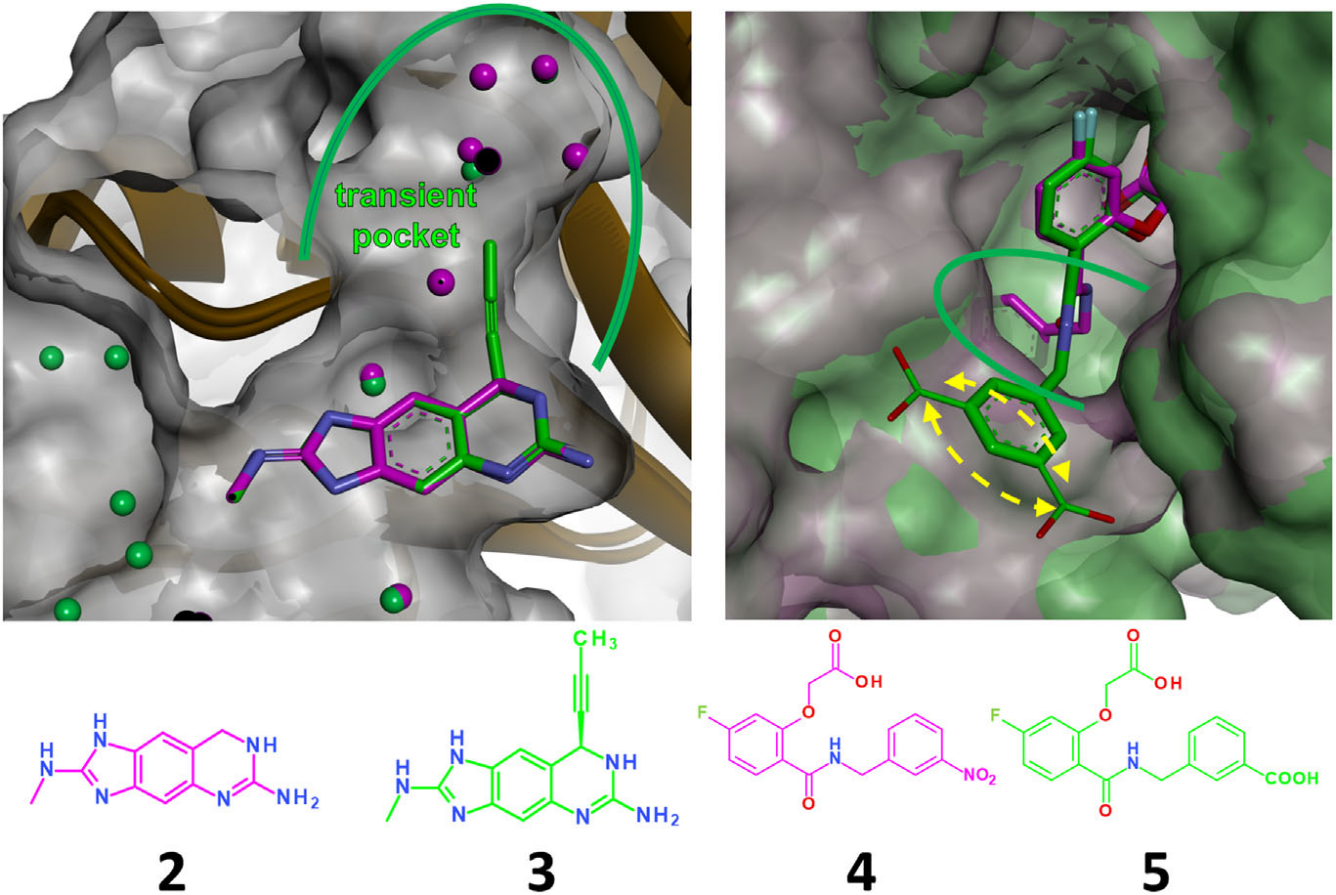
Left: Crystal structures of the enzyme tRNA guanine transglycosylase (TGT) show that the two ligands **2** and **3** open a transient pocket. The smaller ligand **3** (magenta carbon atoms) leaves the transient pocket unoccupied and several water molecules are found in the pocket (magenta spheres). The larger ligand **2** (green) orients its hydrophobic propyn‐1‐yl substituent into the transient pocket and displaces a water molecule compared with **3** (green spheres). Nevertheless, both ligands show very similar binding affinity. Right: Crystal structures of aldose reductase with the congeneric ligands **4** (NO_2_ substituent, magenta) and **5** (COOH substituent, green). Both ligands show deviating binding modes. The 1000‐times more potent **4** (protein gray surface) opens a transient pocket and places its nitro‐benzyl group in the transient pocket. The less potent **5** leaves the pocket sealed (protein surface green, the magenta ligand **4** would formally clash with the green surface) and binds outside on the protein surface. Its carboxy‐benzyl substituent is found scattered over two orientations (indicated in yellow). The mutated variants exhibit very similar geometry, but remarkably also the carboxy derivative **5** prefers binding to the open transient pocket without improving its weak potency. The NO_2_ derivative **4** binds to the variants with a 100‐times reduced affinity compared to its potent binding to the wildtype.

We detected transient pockets that can open in response to ligand binding in tRNA guanine transglycosylase (TGT) [39] and aldose reductase [40, 41] (Figure 4). For the transglycosylase, the transient pocket was captured in a fragment‐bound complex. The fragment does not occupy the pocket, which remains open and water‐solvated. A ligand with a well‐designed hydrophobic propyn‐1‐yl substituent (**2**) binds to the transient pocket and replaces some of the water molecules. However, the binding affinity does not improve compared to an isostructural ligand (**3**) that exhibits the same parent scaffold but lacks the side chain. This truncated ligand binds identically to the protein and shows nearly the same affinity (Figure 4, left). In this example, replacing the water molecules with the attached side chain in the transient pocket nullifies the expected affinity improvement by filling the open transient pocket with a hydrophobic side chain.

We studied the binding of two congeneric aldose reductase inhibitors (**4**, **5**) that differ only in their terminal substituent: a carboxylate or a nitro group (Figure 4, right). Although the two ligands appear to be isostructural, they have a binding affinity that differs by a factor of 1000, and they adopt different binding modes. The nitro derivative opens a transient pocket, while the carboxylate analog leaves the pocket closed and binds to the protein surface outside the pocket. Molecular dynamics (MD) simulations suggest that the nitro derivative follows an induced fit mechanism, opening the pocket as it approaches the protein surface. The pocket closes with a leucine‐to‐phenylalanine gatekeeper contact. Mutational studies revealed an unexpected result. Replacing the leucine side chain with a much shorter alanine or glycine side chains resulted in a 100‐fold drop in affinity for the nitro derivative **4**, yet its binding to the open transient pocket remained unchanged. Conversely, the carboxylate derivative **5** exhibits an affinity for the mutated variants that remains nearly unchanged, though the binding mode to the open pocket becomes favored. Structures with a smaller ligand suggest that the mutated variants have a pocket that is due to the smaller size of the amino acid side chains already partially open and that becomes increasingly flooded with water molecules. Clearly, the nitro derivative, which opens the transient pocket in the wild type and likely follows an induced fit mechanism, loses its ability to bind to a previously unsolvated transient pocket. When binding to the variants that show a partly open and water‐preflooded transient pocket, the nitro derivative must now pay the price for desolating the pocket. Consequently, it loses affinity. In contrast, the carboxylate ligand benefits from preflooding as its negatively charged carboxylate group can interact with the water molecules, thereby stabilizing intermediate steps of the binding process to the transient pocket. However, it does not increase affinity because it must also desolvate the pocket.

### 1.7 Ligand Solvation Prior to Protein Binding Can Be Decisive

**FIGURE 5:**
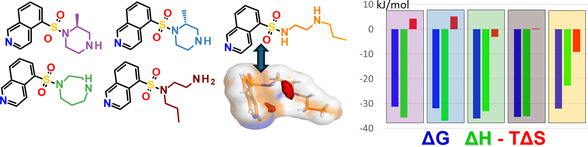
Remarkably, the ligand with the open‐chain substituent (shown in orange, upper right row) is the most flexible ligand in the series. Accordingly, its binding should be entropically unfavorable, as it loses the most internal degrees of freedom. Unexpectedly, it exhibits the most favorable entropic binding profile (most right, red column on the orange‐colored background). This is due to its ability to tightly capture water molecules in a back‐folded conformation in aqueous solution prior to protein binding. The two preferred sites are indicated in red (center, lower row). The release of these fixed water molecules dominates this ligand's overall entropically favored signature.

The latter example clearly demonstrates the influence of water solvation on ligand binding. Additionally, prior to protein binding, ligands in a congeneric series can differ due to solvation effects [42]. For example, one member of a series of substituted fasudil‐type inhibitors (ligand with orange‐colored sidechain) that bind to protein kinase A adopts a back‐folded conformation in the bulk water phase (Figure 5). This conformation captures a water molecule so tightly that its release upon protein binding determines the entire binding signature. Paradoxically, despite being the most flexible member of the series, this ligand exhibits the most entropically favored binding profile. The opposite would be expected. The most flexible ligand should sacrifice the largest number of internal degrees of freedom and thus should exhibit an entropically unfavorable profile. In this case, however, the water molecules released from the back‐folded conformer into the bulk water phase overcompensate for the entropic loss of internal degrees of freedom.

## 2 Conclusion and Outlook

Let's go back to the initial question: Have we come any closer to understanding the role of water molecules in drug discovery? Yes, somewhat. We have seen many individual cases that would have remained unclear without a detailed analysis of the impact of water. Obviously, nature makes use of water molecules in many ways in protein‐ligand binding. For example, we have seen that pocket solvation can govern substrate recognition and binding by adjusting the residual solvation of a binding pocket. Preflooding of pockets is an important factor in fine‐tuning the binding potency of ligands, especially in transient pockets. Perfection of the residual surface‐solvation network around portions of the ligand exposed from a pocket into the surrounding bulk water phase can enhance affinity and contribute to selectivity discrimination. Ligand promiscuity can be controlled by filling gaps resulting from the volume differences of substrate molecules by incorporating water molecules into unoccupied interstitial pocket sites. The enzyme TGT (see above) recognizes two substrates, guanine and preQ1. The latter is extended by a methylene amino group. Both substrates are bound at the same site through a peptide flip and the incorporation of an interstitial water molecule in case of guanine [43]. Similarly, the oligopeptide‐binding protein OppA binds a broad range of amino acids by incorporating or releasing water molecules. This protein is a real master at making use of the water gap‐filling property [44]. Due to its bifunctionality as both a hydrogen bond donor and acceptor, water distinguishes between amino acid replacements in related proteins that recognize different ligands and perform different functions within organisms. Nuclear hormone receptors or phosphodiesterases are good examples [45, 46]. The above‐mentioned enzyme TGT is a glycosylase that orients two facing aspartate residues into the binding pocket. In the uncomplexed state, a network of water molecules binds between the negatively charged aspartates, likely buffering the accumulation of negative charges. This fact must be considered when designing potent ligands that cross the region between the two aspartates [47]. The enzymatic mechanism in TGT requires water as a co‐substrate to accept and deliver protons. Accordingly, the formation of a hydroxide and an oxonium ion as byproducts of the reaction is assumed to cause the irreversibility of the enzymatic reaction, as the mutual neutralization of these two ions in the bulk phase efficiently removes them from equilibrium [48]. These are only a few selected examples, but the list could be expanded endlessly. This makes it quite challenging to provide medicinal chemists with a set of rules that guide their considerations regarding the impact of water. Nevertheless, we must continue this type of analysis to develop a catalog of rules that can support medicinal chemists in selecting the best ligand optimization strategy in the future, as neglecting the influence of water molecules is clearly not an option.

## Funding

This work was supported by the European Research Council (No. 268145 DrugProfilBind).

## Conflicts of Interest

The author declares no conflicts of interest.

## Data Availability

The data that support the findings of this study are openly available in Protein Data Bank at https://www.rcsb.org/.
